# Digital Behavior Change Interventions for Younger Children With Chronic Health Conditions: Systematic Review

**DOI:** 10.2196/16924

**Published:** 2020-07-31

**Authors:** Amberly Brigden, Emma Anderson, Catherine Linney, Richard Morris, Roxanne Parslow, Teona Serafimova, Lucie Smith, Emily Briggs, Maria Loades, Esther Crawley

**Affiliations:** 1 Centre for Academic Child Health, Bristol Medical School University of Bristol Bristol United Kingdom; 2 Bristol Medical School University of Bristol Bristol United Kingdom; 3 Department of Psychology University of Bath Bath United Kingdom

**Keywords:** mobile phone, mHealth, mobile health, eHealth, electronic health, digital health, behavior, pediatrics, chronic illness, systematic review

## Abstract

**Background:**

The prevalence of chronic health conditions in childhood is increasing, and behavioral interventions can support the management of these conditions. Compared with face-to-face treatment, the use of digital interventions may be more cost-effective, appealing, and accessible, but there has been inadequate attention to their use with younger populations (children aged 5-12 years).

**Objective:**

This systematic review aims to (1) identify effective digital interventions, (2) report the characteristics of promising interventions, and (3) describe the user’s experience of the digital intervention.

**Methods:**

A total of 4 databases were searched (Excerpta Medica Database [EMBASE], PsycINFO, Medical Literature Analysis and Retrieval System Online [MEDLINE], and the Cochrane Library) between January 2014 and January 2019. The inclusion criteria for studies were as follows: (1) children aged between 5 and 12 years, (2) interventions for behavior change, (3) randomized controlled trials, (4) digital interventions, and (5) chronic health conditions. Two researchers independently double reviewed papers to assess eligibility, extract data, and assess quality.

**Results:**

Searches run in the databases identified 2643 papers. We identified 17 eligible interventions. The most promising interventions (having a beneficial effect and low risk of bias) were 3 targeting overweight or obesity, using exergaming or social media, and 2 for anxiety, using web-based cognitive behavioral therapy (CBT). Characteristics of promising interventions included gaming features, therapist support, and parental involvement. Most were purely behavioral interventions (rather than CBT or third wave), typically using the behavior change techniques (BCTs) *feedback and monitoring*, *shaping knowledge*, *repetition and substitution*, and *reward*. Three papers included qualitative data on the user’s experience. We developed the following themes: *parental involvement*, *connection with a health professional is important for engagement*, *technological affordances and barriers*, and *child-centered design*.

**Conclusions:**

Of the 17 eligible interventions, digital interventions for anxiety and overweight or obesity had the greatest promise. Using qualitative methods during digital intervention development and evaluation may lead to more meaningful, usable, feasible, and engaging interventions, especially for this underresearched younger population. The following characteristics could be considered when developing digital interventions for younger children: involvement of parents, gaming features, additional therapist support, behavioral (rather than cognitive) approaches, and particular BCTs (*feedback and monitoring*, *shaping knowledge*, *repetition and substitution*, and *reward*). This review suggests a model for improving the conceptualization and reporting of behavioral interventions involving children and parents.

## Introduction

### Background

The prevalence of chronic health conditions in childhood is increasing [1,2]. Chronic health conditions are defined as “any physical, emotional, or mental condition that prevented him or her from attending school regularly, doing regular school work, or doing usual childhood activities or that required frequent attention or treatment from a doctor or other health professional, regular use of any medication, or use of special equipment”[3].

Behavioral interventions can support the treatment and management of chronic health conditions and can be effective in improving symptom management, reducing physical disability, and improving mental health [4-6]. These outcomes are particularly important in childhood because they have implications for children across their lifespan [7-13]. Behavioral or behavior change interventions are sets of techniques that aim to change health behaviors [14]. For children with long-term health conditions, these interventions typically focus on adherence to medical treatment, education about the medical condition, and improving aspects of medical care [15]. A specific example is the management of diabetes via behavioral intervention; glycemic control can be improved by encouraging behaviors of blood glucose monitoring, selection of healthy food choices, attendance at routine clinical appointments, and adherence to insulin therapy or other medications [16]. Improving the management of chronic health conditions at an early age can lead to immediate health improvements, but it also lays the foundations for health across the lifespan of the patient [7]. As such, it is important that younger children (and their families) are supported to improve understanding of their condition and develop self-management skills [17].

Digital interventions can deliver behavior change interventions using mobile phones, smartphones, portable computers, desktop computers, the internet, wearable technology, and television [18]. This is an emerging and rapidly developing field of research, and the potential advantages include increased cost-effectiveness, anonymity for users, appeal to younger people, and the ability for recipients to access interventions anywhere and at their own pace [19-22]. There is a growing body of evidence to suggest that digital interventions are potentially effective for adults and adolescents with chronic health conditions; they have beneficial effects on improving knowledge, self-management, self-care, quality of life, medication use, symptom control, and health service utilization [23-30]. However, there are some potential disadvantages that may affect the uptake, attrition, and efficacy of interventions. Some individuals may not be able to access the intervention because of technical issues, illiteracy, or the cost involved in obtaining the devices. Negative attitudes toward technology may also create barriers to use, and this includes concerns about data security. A lack of strong therapeutic relationships may discourage users and reduce engagement and efficacy of interventions. These potential disadvantages [31,32] should be carefully considered when planning and designing digital interventions. Furthermore, there are limitations with the evidence base for digital interventions, with systematic reviews highlighting the need for clearer reporting and higher quality research [23-29].

Despite the increasing availability of digital interventions and a growing body of evidence for adults and adolescents, there has been inadequate attention to designing and delivering these interventions to children. Children have different developmental characteristics and needs, and the developmental stage of children should be considered when designing interventions [33,34]. To the best of our knowledge, there are no systematic reviews that specifically investigate digital interventions for the management of chronic health conditions in children (aged <13 years). Furthermore, reviews investigating digital interventions for young people with chronic health conditions typically do not include children aged below 10 years [35], or, if they do, only a minority of the interventions included in the reviews include children aged below 13 years [15,36-38], recognizing that there are “fewer interventions targeting…the extreme pediatric age ranges of early childhood and emerging adulthood” [16]. The reviews spanning childhood and adolescence note important differences between these age groups. Three separate reviews of internet and computer-based cognitive behavioral therapy (CBT) for mental health problems found different treatment effects for older and younger children. The reviews found some positive effects for adolescents, but concluded that treatment effects were smaller or more uncertain for younger children [36,37,39]. Similarly, a review of electronic health interventions for young people with long-term physical conditions concluded that effectiveness was uncertain at this time, especially in children aged <10 years [15]. One review acknowledged, “we could not take the developmental stage of the patients…into account. As evidence is mounting, this issue should be addressed in future trials” [17].

Therefore, this review aimed to explore digital interventions for the management of chronic health conditions in children aged between 5 and 12 years.

Behavior change interventions are often complex [40], which can pose a challenge when synthesizing the effects of these interventions [41]. Advances in behavioral science have provided taxonomies and coding systems that help identify specific characteristics or *active ingredients* associated with effective interventions [42]. This includes the behavior change techniques (BCTs) taxonomy [43], which presents 93 discrete BCTs, “observable, replicable and irreducible component of an intervention designed to alter or redirect causal processes that regulate behavior”[43]. In addition to understanding what is being delivered (BCTs), it is important to understand how the content is delivered; this can be categorized using the mode of delivery taxonomy [18]. Identifying the theoretical underpinnings is possible with a coding frame [44]. Using these BCTs, mode of delivery and theory taxonomies in systematic reviews may result in more optimal evidence syntheses and health care practice recommendations [41].

### Objectives

This systematic review aimed to investigate digital interventions for the management of chronic health conditions in children aged between 5 and 12 years. We used an inclusive definition of chronic health conditions that included both physical and mental health. Conceptually, behavioral interventions for physical and mental health conditions are the same; they are designed to change the child’s behavior to improve the clinical outcome. Furthermore, there is a strong overlap between physical and mental conditions; comorbidity of physical and mental health conditions is common [45], and many conditions involve both mental and physical health issues (eg, chronic fatigue syndrome or myalgic encephalomyelitis, pain, and obesity), thus developing integrated approaches toward mental and physical health is increasingly becoming a priority [46]. In this review, we aimed to answer the following questions: (1) Which of these interventions are effective in promoting behavior change for the management of the chronic health condition? (2) What are the characteristics of effective interventions, considering the following: recipients, what is being delivered (BCTs), how this content is being delivered (the mode of delivery), the theoretical basis, and the modality of the intervention? and (3) What are the users’ experiences of the digital intervention?

## Methods

### Registration

The review was prospectively registered in the Prospective Register of Systematic Reviews (PROSPERO) database.

### Search Strategy

We carried out a systematic search of relevant databases: Excerpta Medica Database (EMBASE), PsycINFO, Medical Literature Analysis and Retrieval System Online (MEDLINE), and the Cochrane Library (January 2019). The search strategy included keywords and Medical Subject Headings (MeSH) for (1) children aged between 5 and 12 years, (2) behavior change, (3) randomized controlled trials (RCTs), (4) digital interventions, and (5) chronic health conditions (we used a mixture of generic terms, ie, “Chronic disease,” and also search-specific terms, informed by the most common chronic illness in childhood; Multimedia Appendix 1) [47].

### Screening

To be included in this review, studies had to fulfill the following criteria:

1. Include children aged between 5 and 12 years (this review aimed to examine digital interventions for children in the developmental stages of middle childhood).
2. Include children with a chronic health condition, excluding those with developmental delays.
3. Investigate a digital intervention to promote behavior change. Digital interventions included those delivered via internet (static or interactive websites, automated emails, or web-based apps), personal computers (PCs; eg, PC videogames), social media, mobile phones (automated phone calls or short text messages), or smartphones (mobile websites or smartphone apps). These may be stand-alone interventions or guided (eg, therapist supported).
4. Compare the digital intervention with any comparator.
5. Have an RCT study design (RCTs are considered the gold standard for judging the benefits of treatments [48], and including RCTs only allowed us to focus on the interventions most likely to be adopted into clinical care).
6. Published in peer-reviewed journals and available in English.
7. Published between 2014 and January 2019. We chose a 5-year time frame because of the rapid pace of digital interventions [49], indicating that older interventions were not likely to be relevant.

Titles and abstracts (stage 1) and full-text papers (stage 2) were independently double screened against the inclusion and exclusion criteria using the data management platform Rayyan (stage 1) and Covidence (stage 2). AB screened all papers, and CL, LS, and EB were responsible for the independent second screening. Reasons for exclusion were recorded at stage 2. Discrepancies at both stages were discussed and resolved in meetings by the reviewers. Papers were tracked using the Preferred Reporting Items for Systematic Reviews and Meta-Analyses (PRISMA) flow diagram [50].

### Data Extraction and Synthesis

For data extraction, papers were reviewed independently by 2 researchers and conflicts were resolved in regular meetings (AB reviewed all papers, and CL, LS, and EB were responsible for an independent second review). Two researchers independently coded BCTs (EA and AB, a health psychologist and health psychology trainee, respectively). We extracted information that allowed us to answer the 2 primary research questions, as described in Table 1. If the full text did not contain the information needed, we made 2 attempts to contact the authors by email.

Due to the clinical and methodological heterogeneity, we synthesized data using narrative synthesis [51,52] to answer our research questions. We reported effectiveness based on whether interventions were deemed as *very promising*, *quite promising*, *possibly promising*, *nonpromising,* or *unable to assess effectiveness*, determined by change in the outcomes and the quality of the science (Table 1 defines these categories).

**Table 1:** Data extraction.

Data extraction category	Details extracted
Population	Age: the age range of the population, at the time of entry into the study. Ages were then grouped by UK school *key stage* categories [53]: 5-7 years, corresponding to key stage 1. 8-11 years, corresponding to key stage 2. 12 years, corresponding to key stage 3. Chronic health condition: the chronic health condition that the intervention was designed to target.
Overview of intervention	Overview of aims: the overview of the aims of the RCT^a^. Overview of intervention: an overview of the digital component of the intervention and, if applicable, other key components. Overview of comparator: an overview of the comparator arm or arms.
Aim 1: effectiveness	Overview of *promise*: *promise* was based on the beneficial effects of the intervention and the quality of the study (risk of bias). To determine the beneficial effects of the intervention, we looked at the CIs of the mean difference from baseline to follow-up between the intervention and control group, considering the behavioral outcomes and the primary outcome (or outcomes). We developed 5 categories: Interventions were deemed *very promising* where there were beneficial effects of the intervention on both the primary outcome *and* at least one behavior change outcome, and the evidence was judged as having a low risk of bias. Interventions were deemed *quite promising* where there were beneficial effects of the intervention on the primary outcome *or* at least one behavior change outcome (but not both), and the evidence was judged as having a low risk of bias, or some concerns. Interventions were deemed *possibly promising* where there were beneficial effects of the intervention on the primary outcome and or behavior change outcome (or outcomes), *but* the study was deemed to have a high risk of bias. Interventions were deemed *nonpromising* where there were *no* beneficial effects of the interventions on either the primary outcome or behavior change outcomes. Interventions were put in the category *unable to assess effectiveness* where there were *no* effectiveness data available for the primary outcome or behavior change outcomes, for example, if the paper was a pilot or feasibility RCT. The direction and size of the effect [51] was extracted for behavioral and primary outcomes, and the following was reported: Summary of the effect of the intervention compared with the control. Statistic comparing the change in the intervention group and control group from baseline to final follow-up. Where sufficient information was available comprising either SDs and numbers of participants, or SEs, we calculated the net mean difference (difference in mean changes), with 95% CI and *P* value. Where possible, this was interpreted in the context of the authors’ judgment of clinically significant effects. If available, we reported the adjusted mean difference (adjusted for baseline measures) as this is the accepted best method. Outcome measure: all behavioral outcomes were extracted, as exploring the effect of the intervention on behavior change was the primary aim of this review. The primary outcome was also extracted as this is the main determinant of whether the study is considered a *success* or a *failure* [54]. For each outcome we extracted: the behavior and or primary outcome how this was measured the final time point. Adverse events: health interventions carry some risk of harm. Systematic reviews should minimize bias toward favoring an intervention by assessing adverse effects alongside beneficial effects [55]. Data on adverse events associated with the intervention were extracted.
Aim 2: characteristics of promising interventions	The following data were extracted from very promising, quite promising, and possibly promising interventions: Recipients: whether the intervention was delivered directly to the child, via a parent-proxy or both. Intervention techniques: intervention techniques refer to what is being delivered, the content or *active ingredients* of an intervention. The behavior change techniques taxonomy provides a standardized method of classifying intervention content [43]. This taxonomy consists of 93 behavior change techniques, in 16 groupings. We coded interventions using the 16 groupings. We coded whether each BCT identified was delivered to the parent or the child and whether it featured in the digital or human component. Digital mode of delivery: intervention mode of delivery refers to how the content is delivered. We categorized mode of delivery, based on elements of the mode of delivery Taxonomy [18]: *Tailored or generic:* tailored interventions change the content of the text, images, recommendation, messages, etc based on the individual needs of the user. *Guided or stand-alone:* guided interventions are delivered with some form of support by a professional or coach, either with human contact or electronic means (eg, email or telephone calls). *Interactive techniques*: these include play, arts, story, and or game-based techniques. Theoretical basis: whether a named theory of behavior or behavior change was explicitly mentioned in the Abstract, Introduction, or Methods section [44]. Modality: the intervention modality, coded as either a first, second, or third wave intervention. *First wave* interventions are purely behavioral, based on the theory that all behaviors are learned (through classical and operant conditioning) [56], and that maladaptive behaviors can be changed using principles such as reinforcement, modeling, graded tasks and habit formation [43]. *Second wave* refers to cognitive behavioral interventions, based on the principle that thoughts, feelings, physical sensations and actions are interconnected; individuals are supported to identify negative or unhelpful patterns in their cognitions, emotions, behaviors, physical sensations and supported to adopt more adaptive patterns [57]. *Third wave* interventions are characterized by techniques such as metacognition, acceptance, mindfulness, compassion and spirituality [56].
Aim 3: The users’ experience of the digital intervention	Qualitative analysis: two researchers independently reviewed all eligible papers and identified those that included qualitative data about the users’ experience of the digital intervention. Qualitative data were extracted, compared, and summarized into themes.

^a^RCT: randomized controlled trial.

### Quality Assessments

As all studies in this review were RCTs, the Cochrane risk of bias tool for randomized trials (RoB 2.0) [58] was used to assess the scientific quality of each study. Two researchers reviewed each paper, and the researchers then compared their quality assessment and resolved conflicts (AB reviewed all papers, and ML, LS, and EB were responsible for an independent second review). Following this, each paper was given a score of either low risk of bias, some concerns, or high risk of bias. Where available, we reviewed trial registries and published protocols. If needed, we also requested further information from the authors, including statistical analysis plans.

## Results

### Literature Search and Selection of Studies

**Figure 1:**
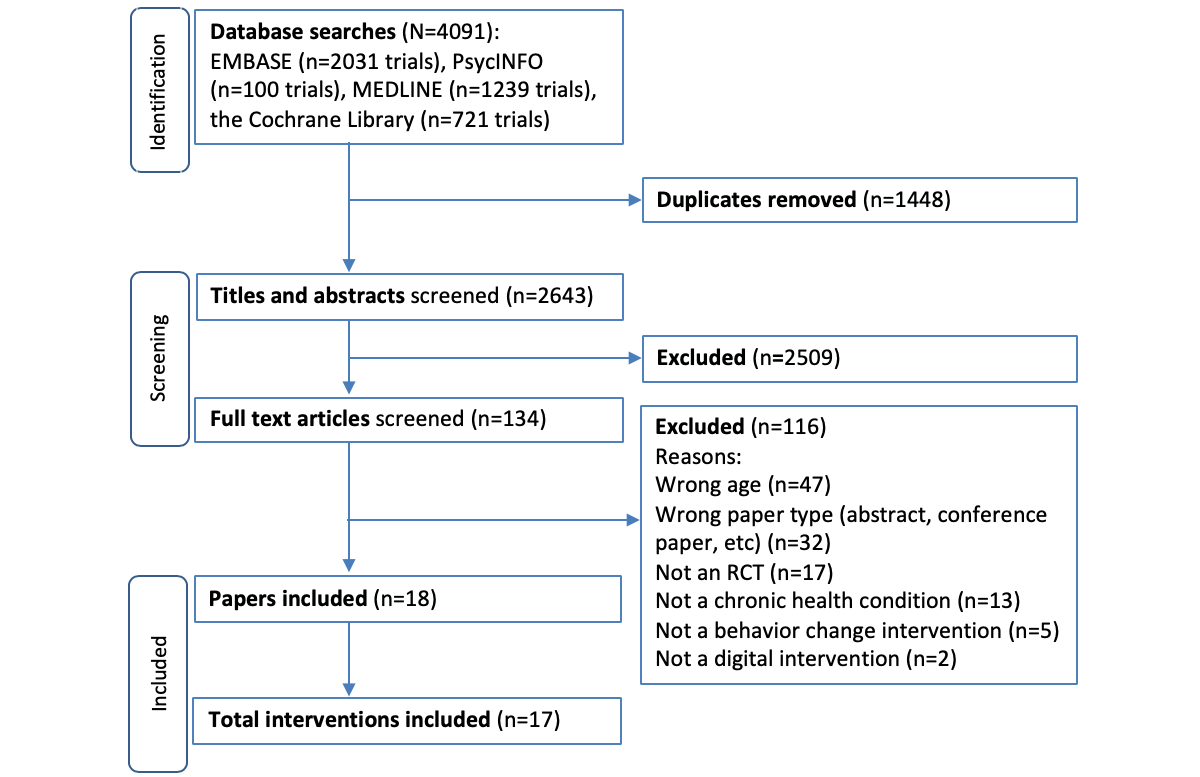
PRISMA flow diagram. RCT: randomized controlled trial.

After deduplication, 2643 papers were identified from the database searches, of which 18 papers were identified as eligible for inclusion. Two of these papers reported on the same intervention; therefore, we identified 17 digital interventions for the management of chronic health conditions in children aged between 5 and 12 years. Figure 1 displays the PRISMA diagram.

### Population Characteristics

The digital interventions targeted a range of chronic health conditions, including overweight or obesity (n=7), anxiety and preoperative anxiety (n=3), cerebral palsy (n=3), attention-deficit/hyperactivity disorder (ADHD; n=1), type 1 diabetes (n=1), asthma (n=1), and social-emotional problems (n=1). All the interventions included children of key stage 2 age (8-11 years), 13 included children of key stage 3 age (12 years), and 9 included children of key stage 1 age (5-7 years).

### Aim 1: Which Digital Interventions Are Effective in Promoting Behavior Change for the Management of the Chronic Health Condition?

Table 2 details the characteristics of the population, intervention, and outcome data (Multimedia Appendix 2). This is presented by their potential effectiveness, based on outcomes and quality assessment (Multimedia Appendix 3).

No interventions were identified as *very promising*.

A total of 5 interventions were identified as *quite promising*; 3 of these were interventions targeting overweight or obesity. An intervention involving exergaming used Kinect and Xbox with additional components of Fitbit step count monitoring and parent-child telehealth sessions with a fitness coach. Compared with the control group, the intervention group showed an increase of 11.4 min of moderate-to-vigorous physical activity (MVPA) per day (95% CI 2.25- 20.55) at 6 months. However, there was no clear evidence of effect on the primary outcome; the reduction in BMI Z-scores was −0.08 (95% CI −0.16 to 0.003) at 6 months, which did not reliably meet the authors’ threshold for change (−0.09). An exergaming plus a family-based pediatric weight management program led to an increase of 8.0 (95% CI 0.5-15.4) min of MVPA per day at 4 months, with the trial powered to detect a change of 4.0 to 4.6 min of MVPA per day (MVPA was the primary and behavior change outcome) [54]. A further trial involved 4 training units (2 face-to-face and 2 via Facebook) plus weekly support through a parental WhatsApp group for 12 weeks. This led to a decrease in BMI Z-scores of 0.14 (95% CI −0.28 to −0.003) at 6 months, with the trial powered to detect a decrease of 0.24 (no behavior change outcome was available). The third and fourth *quite promising* interventions were both internet-delivered CBT for children with anxiety disorders, both offering completely web-based modules for parents and children in conjunction with web-based therapist contact [60,63,64]. Both led to an improvement in anxiety as assessed by the clinician severity rating, equating to an estimated change of −077 (95% CI −1.15 to −0.40) at 3 months [60] and −1.16 (95% CI −1.55 to −0.77) at 10 weeks [63].

**Table 2:** Data on population, interventions, and effectiveness of behavior change outcomes and primary outcomes, grouped by intervention promise.

Categories and reference		Condition; age	Overview of intervention/recipients	Recipients	Behavior change outcome	Primary outcome (if different)
**Quite promising**						
	Ahmad et al (2018) [59]	Overweight or obesity; 8-11 years	Digital component: 2 training units delivered weekly via Facebook. Weekly 1-hour sessions using a parents’ WhatsApp group that lasted for 12 weeks. In the WhatsApp group, the researchers posted key information and skills, responded to parent queries, and provided feedback on the adiposity progress of the children. Parents were encouraged to interact with the group WhatsApp group; additional component: 2 half-day face-to-face training sessions	Digital component: parent only; face-to-face component: parent and child	Healthy lifestyle behaviors, children’s eating, physical activity, and screen time: effectiveness data not reported	BMI Z-score: the intervention group had a reduced BMI Z-score compared with the control. Net mean difference^a^=−0.14 (95% CI −0.278 to −0.003; *P*=.05)
	Jolstedt et al (2018) [60]	Anxiety; 8-12 years	Digital component: ICBT^b^. A web-based program with 12 modules delivered over 12 weeks, consisting of texts, films, illustrations, and exercises and focused mainly on exposure therapy. Limited weekly asynchronous support from a clinician to encourage families to engage in the program. Twelve parent-directed modules, covering parental behaviors, which can maintain anxiety and how to best support their child. Parents helped children with the child-directed modules	Digital component: parent and child; face-to-face component: parent and child	None reported	Anxiety (CSR)^c^: Participants allocated to ICBT showed improvements on the CSR compared with the control. Net mean difference=0.79 (95% CI 0.42-1.16; *P*=.002); the estimated between-group effect size at 12-weeks posttreatment 0.77 (95% CI 0.40-1.15)
	Staiano et al (2018) [61]	Overweight or obesity; 10-12 years	Digital component: participants were provided a Kinect and Xbox 360 gaming console, 4 exergames, and a Fitbit Zip to wear during the 24-week period. Steps per day were wirelessly uploaded and reviewed by the fitness coach; additional component: telehealth, consisting of the child and parent meeting with a fitness coach over video chat, on a weekly basis for the first 6 weeks and biweekly thereafter	Digital component: child only; face-to-face component: parent and child	Physical activity: the intervention showed an increase in MVPA^d^ compared with the control group; estimated mean difference 11.4 min of MVPA per day (95% CI 2.25-20.55); Dietary habits: there was no evidence of effect	BMI Z-score: there was no clear evidence of effect
	Trost et al (2018) [62]	Overweight or obesity; 8-12 years	Digital component: participants were provided a game console and motion capture device (Xbox and Kinect; Microsoft Corporation) and 2 active sports games. No explicit advice or goals were given to any study participant regarding the use of their active gaming tool; additional component: a comprehensive family-based pediatric weight management program	Digital component: child only; face-to-face component: parent and child	Physical activity: the intervention group exhibited a greater increase in MVPA compared with the control group. Net mean difference=8 min of MVPA per day (SE 3.8; 95% CI 0.5-15.4; *P*=.04)	The primary outcome was physical activity (see the behavioral outcomes column)
	Vigerland et al (2016) [63] and Vigerland et al (2017) [64]	Anxiety; 8-12 years	Digital component: a treatment platform with 11 modules, including reading material, films, animations, illustrations, and exercises. A combined parent-child intervention. Seven parent-directed modules containing information and instructions on how to help their child. Four child-directed modules. Participants had web-based contact with an assigned psychologist or CBT therapist through written messages and written feedback. Three scheduled telephone calls plus additional telephone calls if needed (to increase motivation or problem solve)	Digital component: parent and child; face-to-face component: parent and child	None reported	Anxiety (CSR): the intervention group had a larger improvement on the CSR. Net mean difference=−1.16 (95% CI −0.77 to −1.55)
**Possibly promising**						
	Bul et al (2016) [65]	ADHD; 8-12 years	Digital component: a serious web-based adventure game (Plan-It Commander) developed by health care professionals, researchers, and game experts in collaboration with parents and children with ADHD. A web-based mission-guided game in which principles of behavior therapy and game-based learning were combined. The missions addressed specific skills addressing time management, planning and organizing, and prosocial behavior. Players could access a closed social community (*Space Club*) to stimulate prosocial behavior (eg, helping other players and giving compliments); additional component: treatment as usual	Digital component: child only; face-to-face component: N/A^e^	Time management: the intervention arm showed greater improvements in parent-reported time management skills compared with the control arm. Estimated net mean difference of parent-reported time management=5.98 (95% CI 1.32-10.64) and teacher time management=5.46 (95% CI 1.71-9.20); planning and organizing skills, social skills: no clear evidence of effect	The 3 primary outcomes were: parent-reported time management, parent-reported planning and organization, and parent-reported social skills (see the behavioral outcomes column)
	Hsieh et al (2018) [66]	Cerebral palsy; 5-10 years	Digital component: a PC^f^ gaming platform. The participants stood in front of the platform and viewed a monitor that displayed 1 of a series of simulated tasks, such as hitting ground rats. The gaming platform handle was loaded, 0.5 to 2.5 lb. This PC gaming platform provided trunk movements in 3 directions: horizontal, vertical, and multidirectional trunk movements	Digital component: child only; face-to-face component: child only	None reported	Measures of postural balance: there was clear evidence of effect of the intervention for 2 of the 7 measures
	Wantanakorn et al (2018) [67]	Preoperative anxiety; 5-12 years	Digital component: *Children-Friendly Hospital*, a tablet app for pediatric patients who need bone marrow aspiration procedures. It was used to provide medical information: participants started with the cartoon about the procedure then played matching games and practiced the breathing exercise game to reduce anxiety; additional component: usual bone marrow aspiration procedures	Digital component: child only; face-to-face component: N/A	N/A	Preoperative anxiety: anxiety was lower in the intervention group versus the control group. Estimated difference means=−7.71 (95% CI −14.27 to −1.15)
**Nonpromising**						
	Armstrong et al (2017) [68]	Overweight or obesity; 5-12 years	Digital component: daily mobile text messages, based on motivational interviewing, for 12 weeks. Initial texts encouraged parents to set a health behavior goal. In a reply text, the investigators reinforced the most evidence-based goals for BMI reduction (sugar-sweetened beverage reduction, increased physical activity, eating meals at home, and increased vegetable consumption). Subsequent daily texts prompted parents to self-monitor adherence to the goal. Each week for 12 weeks, parents can choose a new goal or continue working on the present goal; additional component: standard care, including monthly lifestyle counseling visits by a physician and dietician	Digital component: parent only; face-to-face component: parent only	Child nutrition habits, activity habits and screen time: no clear evidence of effect of intervention	BMI Z-score: no clear evidence of effect of intervention
	Christison et al (2016) [69]	Overweight or obesity; 8-12 years	Digital component: The Exergaming for Health Program is a community-based, multifaceted pediatric weight management program including 1 hour of weekly group exergaming; additional component: classroom curriculum	Digital component: child only; face-to-face component: parent and child	Activity levels, sedentary screen time, and diet: no clear evidence of effect of intervention	BMI Z-score: no clear evidence of effect of intervention
	Sanchez et al (2017) [70]	Social-emotional problems; 7-11 years	Digital component: a single-player story-based digital game that requires children to apply specific social-emotional skills to solve social problems encountered in the game. For example, approaching an individual that appears easy to talk to, joining a group at a game in progress, and approaching small groups that appear less easy to talk to. Within each scenario, the player had to make behavioral choices and individualized feedback to choices was given	Digital component: child only; face-to-face component: N/A	Bullying perpetration: no clear evidence of effect of intervention	No primary outcome specified
**Unable to assess effectiveness**						
	Burckhardt et al (2018) [71]	Type 1 diabetes; 8-12 years	Digital component: the Dexcom G5Ò Mobile continuous glucose monitoring system transmitted glucose levels via Bluetooth to a mobile device that generated alerts. This information could be shared via the *cloud* with up to 5 individuals who could remotely monitor the continuous glucose reading in real time along with the possibility to use individualized alerts. Participants were able to see their sensor glucose levels in real time	Digital component: child only; face-to-face component: N/A	Pilot study (n=14)	No primary outcome specified
	Fiks et al (2015) [72]	Asthma; 6-12 years	Digital component: the features of MyAsthma include identification of parents’ concerns and goals for asthma treatment; monthly tracking of symptoms, medication side effects, and progress toward goals; educational content; and access to the child’s asthma care plan. Parents were encouraged with email reminders to complete monthly portal surveys with input from their affected child. In response to these surveys, families and clinicians received guideline-based decision support that directed them to speak to one another if asthma was not well controlled or if there were side effects, or to continue current therapy	Digital component: parent only; face-to-face component: parent only	Only acceptability or feasibility data	Only acceptability or feasibility data
	Hamilton-Shield et al (2014) [73]	Overweight or obesity; 5-11 years	Digital component: Mandolean teaches patients how to eat and recognize satiety. The patient puts a measured portion of food determined by a therapist on the Mandolean (scales and computer), which records and displays, in real-time graphics, the removal of food from the plate as the patient eats. This is compared with a preset eating line and deviation from the training line by eating too quickly or slowly elicits a spoken request from Mandolean to slow down or eat faster. The patient rates their level of satiety, which appears as a dot on screen yielding a *development of satiety* curve allowing comparison of the development of fullness with a *normal* fullness curve again preset on screen. Additional component: standard care comprising of dietary and activity advice by trained practice nurses	Digital component: child only; face-to-face component: parent and child	None of the pilot trial’s objectives were met; thus there were no full trial effectiveness results	BMI Z-score: none of the pilot trial’s objectives were met; thus there were no full trial results
	Kassee et al (2017) [74]	Cerebral palsy; 7-12 years	Digital component: a Nintendo Wii U system, 1 Wii MotionPlus remote controller, 1 Wii Nunchuck, and the Wii Sports Resort game to be played at home. Games were designed to promote higher upper-limb activity. Participants were instructed to play games using their affected hand for at least 40 min each day, 5 days a week for 6 weeks (30 days); additional component: parents supervised and recorded sessions and were asked to encourage the child to use their spastic hand as much as possible	Digital component: child only; face-to-face component: parent and child	Pilot study (n=6)	Pilot study (n=6)
	Preston et al (2016) [75]	Cerebral palsy; 5-12 years	Digital component: computer-assisted arm rehabilitation gaming used at the child’s home. Parents were asked to encourage their children to use the gaming technology for 30 min a day; additional component: a visit at week 3 to offer encouragement and to check the gaming technology system. Usual follow-up treatment	Digital component: child only; face-to-face component: parent and child	None reported	Pilot study (n=15)
	Price et al (2015) [76]	Overweight or obesity; 6-12 years	Digital component: text messages to parents to reinforce telephone health behavior coaching. Text messages to promote behavioral self-monitoring and skills training, focused on behaviors, including limiting fast food and eating fruits and vegetables in place of high-calorie snacks. At the time of a well child care visit, digital alerts were sent to pediatricians designed to identify children with a BMI ≥95th percentile. These contained information on how to monitor and support the child; additional component: Well child visit	Digital component: parent only; face-to-face component: parent only	Only acceptability or feasibility data	Only acceptability or feasibility data

^a^Net mean difference indicates the difference in mean change between the intervention and the control arms of the study.

^b^ICBT: internet-delivered cognitive behavioral therapy.

^c^CSR: clinician severity rating.

^d^MVPA: moderate-to-vigorous physical activity.

^e^N/A: not applicable.

^f^PC: personal computer.

In all, 3 interventions were identified as *possibly promising.* A PC game led to improved balance control in children with cerebral palsy on 2 of the 7 measures of balance at 3 months [66]. An internet-based serious game for ADHD led to an improvement in parent- and teacher-rated time management skills at 5 months, but no evidence of improvement on parent- and teacher-rated planning and organization skills or social skills. A tablet app that included an educational animated video, along with games for distraction and to encourage relaxation or breathing exercises for preoperative anxiety [67], led to reduced anxiety scores on the modified Yale Preoperative Anxiety Scale of −7.71 (95% CI −14.27 to −1.15) immediately after the intervention. Although there was evidence of an effect, these studies were limited in scientific quality. There was a lack of transparency around randomization processes, a combination of nonblinded participants, and the use of self-report measures, and none of these trials were prospectively registered.

Three interventions showed no promise; 2 of these were targeting overweight and obesity, 1 was the exergaming plus classroom curriculum, and the other was motivational interviewing delivered via one-way text messaging [68,69]. Neither lead to improvements in behavioral outcomes (screen time, physical activity, and diet) or the primary outcome (BMI Z-scores) at 6 and 3 months, respectively. The other intervention was a video game for social-emotional problems [70], which did not lead to changes in bullying perpetration behavior at 9 weeks.

Six interventions were pilot studies, and they only reported acceptability or feasibility data [72,73,76] or involved small sample sizes (6, 15, and 14) that were not powered to determine effectiveness [71,74,75]. Of these studies, 3 reported that there were no further plans for investigation [72-74] and 1 reported that a larger, fully powered trial was planned for the future [75]. Information on the remaining 2 studies is unknown [71,76].

Adverse events for each study are reported in Table 3. Three studies reported adverse events; these were not severe and or there were similar numbers in the intervention and control arms. Four studies monitored adverse events and reported that no adverse events occurred during the trial. Most studies (n=10) failed to capture adverse events.

**Table 3:** Summary of adverse events.

References	Details of adverse events
Ahmad et al (2018) [59]	“No adverse events or unintended adverse consequences of the intervention were reported by the participants.”
Armstrong et al (2017) [68]	“We observed no adverse events associated with participation in the text message intervention.”
Bul et al (2016) [65]	“There were 10 adverse events that could be related to the intervention... All adverse events were of mild (n=5) or moderate (n*=*5) severity… Examples of adverse events were pain in the fingers, irritability, and headache. An adverse event was a reason to discontinue the study for only one known participant. This participant did not want to play the game anymore because he could not concentrate during his school activities. Sounds reminded him of the game and this consequently distracted and frustrated him. No serious adverse events were reported.”
Burckhardt et al (2018) [71]	Did not capture adverse events.
Christison et al (2016) [69]	Did not capture adverse events.
Fiks et al (2015) [72]	Did not capture adverse events.
Hamilton-Shield et al (2014) [73]	“There were no adverse events regarded as serious, unexpected or suspected to be related to the study treatment”
Hsieh et al (2018) [66]	Methods: “no adverse effects were expected in participants in the intervention group.” No further details of adverse effects were reported.
Jolstedt et al (2018) [60]	“No severe adverse events were reported in either group... The number of adverse events was similar between the groups.” Total reported adverse events: ICBT^a^ 17 (26%), ICDP (active control) 16 (25%).
Kassee et al (2017) [74]	Did not capture adverse events.
Preston et al (2016) [75]	“No adverse events were reported.”
Price et al (2015) [76]	Did not capture adverse events.
Sanchez et al (2017) [70]	Did not capture adverse events.
Staiano et al (2018) [61]	“Among those randomized to the intervention group, two children reported an injury during gameplay (bruise to the ankle or wrist).” “Two adverse events (bruising) were reported in the GameSquad trial, which is similar to prior exergaming studies reporting minor bruises, hand lacerations and back pain ...”
Trost et al (2018) [62]	Did not capture adverse events.
Vigerland et al (2016) [63] and Vigerland et al (2017) [64]	Did not capture adverse events.
Wantanakorn et al (2018) [67]	Did not capture adverse events.

^a^ICBT: internet-delivered cognitive behavioral therapy.

### Aim 2: What Are the Characteristics (Active Ingredients) of Effective Interventions?

We considered the 8 interventions that were classified as promising, quite promising, and possibly promising to represent *promising interventions*.

### Recipients

A total of 7 of the 8 interventions had a digital component for the child, and all the interventions involved the child in some capacity (either digital or human component). In all, 5 of the 8 interventions involved the parent in some capacity (either digital or human component).

### What Is Being Delivered: BCTs

Table 4 provides the definitions of the BCTs, and Table 5 provides a summary of the BCTs used in promising interventions.

All the promising interventions used more than one BCT. Digital components for the child typically included techniques coded into the following BCT categories: *feedback and monitoring*, *shaping knowledge*, *repetition and substitution*, and *reward and threat* (we note that none used *threat*, but this is the overarching BCT taxonomy category label). Digital components for the parent typically included *goals and planning*, *social support*, and *natural consequences*.

The most promising interventions were for overweight or obesity (3 studies) and anxiety (2 studies). All 3 of the promising overweight or obesity interventions included a face-to-face component for both the parent and the child. Two interventions included a digital component for the child, both using the BCT repetition and substitution. Only 1 intervention had a digital component for the parent.

Both promising anxiety interventions included digital and face-face elements, all of which involved both the child and the parent. Both interventions used the following BCTs in the digital component: goals and planning (child and parent components), shaping knowledge (child and parent components), feedback and monitoring (parent component), and associations (child component).

We acknowledge that there may have been more BCTs included in the intervention; however, we were unable to code these as they were not explicitly reported in the paper. Furthermore, it was often unclear as to whether the BCT was delivered to the parent or the child and by what means it was planned to take effect. In some cases, we believe that the BCTs were directed at the parent, with the parent then eliciting behavior change in the child. However, none of the papers addressed this level of complexity; they did not describe this mechanism of change nor did they include a parent behavior change outcome measure.

**Table 4:** Definitions of behavior change techniques.

BCT^a^ categories^b^	Definitions
Goals and planning	Includes setting and reviewing goals defined in terms of the behavior (eg, physical activity) or the outcome (eg, weight loss); problem-solving to overcome barriers and or increase facilitators; and detailed action planning of the behavior, considering the context, frequency, duration, and intensity of the behavior
Feedback and monitoring	Includes observing or recording the behavior or the outcome either by the recipient (self-monitoring) or by others; feedback on the performance of the behavior or the outcome
Shaping knowledge	Includes advising how to perform the behavior, the factors that reliably predict performance of the behavior, alternatives to unhealthy behaviors, and how to carry out behavioral experiments
Repetition and substitution	Includes practicing the behavior in a context or at a time when the performance may not be necessary to increase habit and skill; setting easy-to-perform tasks, making them increasingly difficult, but achievable, until the behavior is performed
Reward and threat	Includes using material (eg, money and vouchers) or social (eg, praise) incentives and rewards for the behavior or outcome; informing that future punishment or removal of reward will be a consequence of performance of an unwanted behavior
Social support	Includes advising, arranging, or providing social support (eg, from friends, relatives, colleagues, “buddies,” or staff) for practical and or emotional reasons
Natural consequences	Includes providing information (eg, written, verbal, visual) about the health, social, emotional, or environmental consequences of performing the behavior; using methods to emphasize the consequences
Associations	Includes introducing environmental or social stimulus to prompt or cue behavior; reducing situations in which unwanted behavior can be rewarded; systematic confrontation with a feared stimulus to reduce the response to a later encounter; and presenting a neutral stimulus jointly with a stimulus that already elicits the behavior repeatedly until the neutral stimulus elicits that behavior

^a^BCT: behavioral change techniques.

^b^The study by Michie et al [43] provides a full description of all BCTs.

**Table 5:** Characteristics of promising interventions.

Characteristics			Child recipient–digital component (n=7), n (%)		Child recipient–human component (n=6), n (%)		Parent or caregiver–digital component (n=3), n (%)		Parent or caregiver–human component (n=5), n (%)
**Digital mode of delivery**									
	Tailored	1 (14)		N/A^a^		1 (33)		N/A	
	Guided	5 (71)^b^		N/A		3 (100)^b^		N/A	
	Gaming features	5 (71)^b^		N/A		2 (67)^b^		N/A	
**Behavior change technique**									
	Goals and planning	3 (43)		3 (50)^b^		3 (100)^b^		4 (8)^b^	
	Feedback and monitoring	4 (57)^b^		4 (67)^b^		0 (0)		4 (80)^b^	
	Social support	2 (29)		3 (5)^b^		3 (100)^b^		4 (80)^b^	
	Shaping knowledge	4 (57)^b^		3 (50)^b^		1 (33)		3 (60)^b^	
	Natural consequences	0 (0)		0 (0)		2 (67)^b^		1 (20)	
	Comparison of behavior	2 (29)		2 (33)		1 (33)		1 (20)	
	Associations	2 (29)		1 (17)		1 (33)		1 (20)	
	Repetition and substitution	5 (71)^b^		0 (0)		0 (0)		0 (0)	
	Comparison of outcomes	0 (0)		1 (17)		1 (33)		1 (20)	
	Reward and threat	6 (86)^b^		2 (33)		1 (33)		1 (20)	
	Regulation	1 (14)		0 (0)		0 (0)		0 (0)	
	Antecedents	1 (14)		2 (33)		1 (33)		2 (40)	
	Identity	0 (0)		0 (0)		1 (33)		1 (20)	
	Scheduled consequences	0 (0)		0 (0)		0 (0)		0 (0)	
	Self-belief	0 (0)		1 (17)		1 (33)		2 (40)	
	Covert learning	0 (0)		0 (0)		0 (0)		0 (0)	

^a^N/A: not applicable.

^b^≥50% of interventions using the characteristic.

### How Is the Content Delivered: Mode of Delivery

A total of 5 of the 7 interventions with child digital components used gaming features. All the parent digital components and 5 of the child digital components were guided. In all, 3 digital interventions involving parents and 1 digital intervention for the child were tailored.

### Theoretical Basis

Half of these papers reported the use of theory in the intervention: social cognitive (n=2) and CBT (n=2).

### Modality

A total of 6 of the 8 interventions were first wave (purely behavioral) interventions, and 2 were second wave (cognitive-behavioral) interventions. There were no third wave interventions.

### Aim 3: What Are the Users’ Experience of the Digital Intervention?

Only 3 of the studies included qualitative data on users’ experiences and views of the intervention [68,72,73]. One study evaluated the family experience in a preceding pilot study [77]. A table of the raw qualitative data and themes are available (Multimedia Appendix 4).

### Themes

#### Parental Involvement

Parents talked about the interventions improving their knowledge (“made me more aware”) and shaping their behavior, which in turn led to the child’s behavior change (“it does make me stop him and sit him down and make him eat the breakfast”). Some commented on the problems of parent-led interventions and how a health professional, who is external to the parent-child relationship, is important to encourage the child’s behavior change (“I think some kids will listen to their doctor better than their parents”).

#### Connection With a Health Professional Is Important for Engagement

Digital interventions were seen to facilitate *convenient* communication with a health care professional. There was a desire to share information between parents and clinicians (“It should go back somehow to the paediatrician”) to increase families’ motivation to engage with interventions. The involvement of a health professional was also viewed as important in engaging the child (“I think some kids will listen to their doctor better than their parents”).

#### Technological Affordances and Barriers

Parents commented on the technologies being quick, easy, and possible to integrate into everyday life. However, others commented on practical challenges such as the cost, lack of familiarity, and difficultly to use. Users commented on the fixed nature of the technology, which meant that it was not personalized to their individual preferences or needs (“but I really want to focus on these”) and did not deliver content with ongoing relevance that would maintain engagement over time (“I think enthusiasm’s gone off”).

#### Child-Centered Design

Children commented on some of the interventions being enjoyable (“I like the electronic stuff”). However, in other cases, the material was not understood by the child (“It’s really confusing*”* and “I don’t know how much [child] actually understands”), it was not acceptable to children (“boring” and “annoying”), and they expressed a wish for features such as personalization in the design.

## Discussion

### Principal Findings

To the best of our knowledge, this is the first review to identify effective digital interventions for younger children, report the characteristics of promising interventions, and describe the user’s experience of digital interventions. Of the 17 eligible interventions, we only identified 5 that had a beneficial effect and had a low risk of bias; 3 targeted overweight or obesity, using exergaming or social media with additional human support, and 2 targeted anxiety, using web-based CBT with therapist support.

Characteristics of promising digital interventions included gaming features in the child digital component and having additional therapist support (guided digital interventions). Digital components for the child typically used the BCTs [43] *feedback and monitoring*, *shaping knowledge*, *repetition and substitution*, and *reward*. Most were purely behavioral interventions (first wave), with only a quarter using CBT (second wave) and none using third wave approaches; half of the interventions had a theoretical basis (social cognitive theory and CBT). Over 60% involved the parents in the intervention.

Only 3 papers used qualitative methods to explore the users’ experience of digital intervention. These studies reported the affordances of digital interventions, including ease of use, integration into daily life, and the ability to enhance communication with a health professional. However, a lack of personalization, technical problems, and cost issues posed challenges to families. The qualitative data indicated how the content (eg, language and concepts) and design could be improved for younger users.

### Strengths and Limitations

We included a range of chronic health conditions, which enabled us to review a larger number of interventions and identify patterns or commonalties of promising interventions. Spanning health conditions makes these findings relevant to a wide audience of researchers working in the field of digital interventions. We focused on RCTs because they have the strongest study design and are most likely to be adopted in clinical care [78]. This review focused on the outcomes that were most important to our research question (behavioral outcomes) and most important for that particular study (the primary outcome). It was outside the scope of this paper to review all the possible outcomes, such as health status or symptoms of the disease, quality of life, and knowledge.

Guidance was followed on how to report effectiveness in narrative reviews [51]. We extracted a common statistic to show the size and direction of effect, and where possible, we placed results in the context of clinically meaningful change [79]. Strengths of narrative synthesis include richer exploration of more complex questions, exploring both effectiveness (aim 1) and what “might explain differences in direction and size of effect... how and why interventions have or do not have an effect” (aim 2) [51]. We increased the rigor of presenting characteristics of interventions by using established coding systems and taxonomies for BCTs [43], modality [56], mode of delivery [18], ages [53], and population type [80]. We also considered parental and child components separately, which is important for this younger population.

A limitation of this review is that we only included RCTs. Although observational studies and nonrandomized trials could have provided additional information on the characteristics and effectiveness of digital interventions for this population, we excluded these study designs as they have a greater potential for risk of bias [81]. Although we believe that our search strategy (which included the terms “Randomized Controlled Trial,” “Trial,” and “Clinical Study”) was broad enough to identify different RCT designs, it is possible that we may not have identified some designs specifically used in the evaluation of digital interventions, such as micro randomized trials. We also restricted our search to papers published after 2015. We chose this strategy as digital health is a rapidly changing field, and recently conducted studies are likely to be the most relevant. We excluded studies that included our target age group (5-12 years) but also included older and younger children (eg, 5-18 years). Although it is possible that these studies could have been stratified by age, it was not feasible to contact authors to request these stratified data. As expected, the broad scope of this review led to heterogeneity across studies (in terms of population, intervention, and outcome), meaning formal meta-analysis was not possible; therefore, we selected the most appropriate method, narrative synthesis. Although potential limitations to narrative synthesis include a lack of transparency and reproducibility and being subject to author interpretation [52], we mitigated this by prospectively registering our protocol, with specified outcomes, and following narrative synthesis guidelines [51]. To identify the characteristics of effective interventions, we reviewed both *quite promising* and *possibly promising* interventions and acknowledged that the *possible promising* interventions were of poorer scientific quality. Due to the small number of qualitative studies, we did not conduct full meta-ethnography [82] to synthesize qualitative data, and we did not undertake critical appraisal. However, to increase the transparency of our qualitative summary, we reported the raw data from the papers along with the themes developed by us.

### Implications for Developing, Evaluating, and Implementing Digital Interventions for Children With a Chronic Health Condition

#### Clinical Implications

This review identified promising exergaming and social media interventions for children with obesity or overweight and web-based CBT for children with anxiety. There is potential for these to be implemented in clinical practice with further surveillance, monitoring, and long-term follow-up [40]. These findings are consistent with a previous systematic review that concluded that digital game-based interventions should be considered as methods to promote physical activity among children, but that there is a need for further, high‐quality research that provides more sound evidence about clinical practice and health promotion [83]. This study extends a previous meta-analyses investigating digital interventions for children with anxiety, which concluded that the quality of studies was low (lack of blinding, use of subjective outcome measures, waiting list comparison groups, and relatively small samples) and that the effect is uncertain for younger children [36]. Our review updates this work, identifying 2 interventions with promise. These trials had sample sizes of 131 and 93, and both were prospectively registered trials with prespecified primary outcomes; 1 trial used a blinded outcome assessor for the primary outcome and an active control.

### Implications for Developing and Evaluating Interventions

This work highlights characteristics that may be beneficial when developing digital interventions for younger populations. The finding that purely behavioral interventions (first wave, not including cognitive components) are common in promising interventions is consistent with developmental theory; children tend to be limited to concrete thought [57]. There were fewer CBT (second wave) interventions, possibly because elements of CBT require abstract thinking, which may be beyond the cognitive abilities of children aged <8 years [57]. Similarly, third wave interventions also include abstract concepts such as metacognition. The lack of third wave approaches may also be explained by the fact that this is a relatively new approach for children. As such, concrete interventions focused on behavioral recommendations may be more appropriate [84]. Caregivers are commonly involved in promising interventions. This is also consistent with developmental theory, which highlights the important role of caregivers in structuring the child’s environment and shaping the child’s behavior [84,85]. Gaming features have been used in many promising interventions. Digital games can be adapted to the developmental level and can effectively engage younger users in medical education and treatment, as they are typically more visually oriented, involve appealing exploration, and are perceived as fun [17]. Consistent with the literature, guided interventions were common in promising interventions and have been identified as a moderating factor that can influence therapeutic outcomes and engagement [86,87].

**Figure 2:**
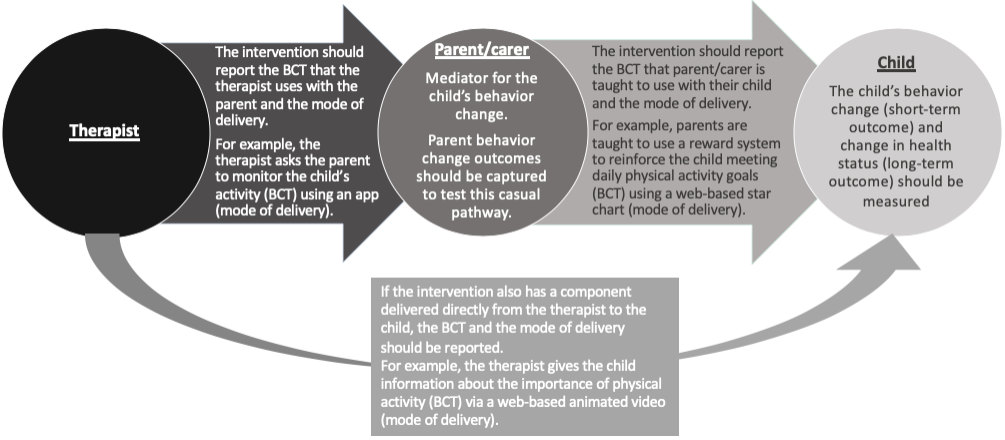
Conceptualising and reporting interventions involving both the parent/caregiver and the child. BCT: behavior change techniques.

Guidelines encourage standardized reporting of interventions to ensure transparency and reproducibility [43,88]. On the basis of our findings, we have developed recommendations for increasing the clarity of interventions with parental involvement. Interventions with both a child and a parent recipient have a complex model of behavior change; it is likely that the therapist aims to shape the behavior of the parent, with the expectation that the parent will change the behavior of the child. Studies in this review failed to explicitly differentiate the BCTs used by the therapist for parental behavior change and the behavior techniques used by the parent for child behavior change. Furthermore, none of the studies in this review captured a parental behavior change outcome measure, when this may be on the causal pathway to the child’s behavior change. This recommendation is consistent with guidelines on process evaluation; outcome measures should be used to test the causal mechanism of the intervention. Figure 2 illustrates the relationship between therapist, parent, and child, detailing our recommendations for how these interventions could be conceptualized and reported.

The low number of promising interventions demonstrates the need to better understand the perspective of those receiving interventions. Few studies have conducted qualitative research to explore the user’s experiences. Qualitative methods, such as the person-based approach [89], base the development and evaluation of digital interventions on an in-depth understanding of the perspectives of the people who will use the intervention. This can lead to interventions that are more meaningful, usable, feasible, and engaging in improving uptake and adherence and maximizing effectiveness [89].

### Conclusions

Of the 17 interventions, we only identified 5 with promise (those with a beneficial effect and low risk of bias). Using qualitative methods during digital intervention development and evaluation may lead to more meaningful, usable, feasible, and engaging interventions, especially for this under-researched younger population. Promising interventions were exergaming and social media for obesity or overweight and a web-based CBT platform for anxiety. We identified characteristics that could be considered when developing digital interventions for younger children: involvement of parents, gaming features, additional therapist support, behavioral (rather than cognitive) approaches, and particular BCTs (*feedback and monitoring*, *shaping knowledge*, *repetition and substitution*, and *reward*). We suggest a model for improving the conceptualization and reporting of behavioral interventions involving children and parents.

## Appendix

Multimedia Appendix 1 Search Strategy.

Multimedia Appendix 2 Full data extraction table.

Multimedia Appendix 3 Summary of risk of bias assessment.

Multimedia Appendix 4 The users experience and views on the digital intervention; raw qualitative data and themes.

## Abbreviations

ADHD: attention-deficit/hyperactivity disorder
BCT: behavior change technique
CBT: cognitive behavioral therapy
MVPA: moderate-to-vigorous physical activity
NIHR: National Institute for Health Research
PC: personal computer
PRISMA: Preferred Reporting Items for Systematic Reviews and Meta-Analyses
RCT: randomized controlled trial
