# Supplementary material for: Digital Behavior Change Interventions for Younger Children With Chronic Health Conditions: Systematic Review
Source: J Med Internet Res. 2020 Jul 31;22(7):e16924. doi: 10.2196/16924 (PMC7428934; doi:10.2196/16924)
Supplement: Multimedia Appendix 1 [file jmir_v22i7e16924_app1.docx]

## Multimedia Appendix 1: Search Strategy

1. Child/
2. child/ or child, preschool/
3. primary school child*.ti,ab,kf.
4. elementary school child*.ti,ab,kf.
5. boy*.ti,ab,kf.
6. girl*.ti,ab,kf.
7. pre-adolescen*.ti,ab,kf.
8. preadolescen*.ti,ab,kf.
9. early life.ti,ab,kf.
10. kindergarten.ti,ab,kf.
11. infant* school.ti,ab,kf.
12. junior school.ti,ab,kf.
13. (young adj child*).ti,ab,kf.
14. (young adj child*).ti,ab,kf.
15. exp behavior therapy/ or biofeedback, psychology/ or cognitive therapy/ or "acceptance and commitment therapy"/ or mindfulness/
16. ((cognitive or cognition) adj3 therap*).ti,ab,kf.
17. CBT.ti,ab,kf.
18. behavio$r* therap*.ti,ab,kf.
19. cogniti* behavio* therap*.ti,ab,kf. 20. behavio$r* intervention*.ti,ab,kf. 21. cogniti* intervention*.ti,ab,kf. 22. Clinical Trial/
20. behavio$r* intervention*.ti,ab,kf.
21. cogniti* intervention*.ti,ab,kf.
22. Clinical Trial/
23. Controlled Clinical Trial/
24. Randomized Controlled Trial/
25. controlled clinical trial.ti,ab,kf.
26. randomi?ed controlled trial.ti,ab,kf.
27. placebo*.ti,ab,kf.
28. trial.ti,ab,kf.
29. (control* adj3 (trial* or study or studies)).ti,ab,kf.
30. ((waitlist* or wait* list* or treatment as usual or TAU) adj3 (control or group)).ti,ab,kf.
31. Clinical Study/
32. exp Cognitive Therapy/
33. intervention*.ti,ab,kf.
34. Cellular Phone.mp. or Cell Phone/
35. SMARTPHONE/
36. Text Messaging/
37. INTERNET/
38. Social Media/
39. Mobile Applications/
40. COMPUTERS/
41. SOFTWARE/
42. Electronic Mail/
43. Video Games/
44. digital.ti,ab,kf.
45. e$mail.ti,ab,kf.
46. television.ti,ab,kf.
47. behavio$r* change.ti,ab,kf.
48. tv.ti,ab,kf.
49. electronic mail.ti,ab,kf.
50. new media.ti,ab,kf.
51. social media.ti,ab,kf.
52. electronic media.ti,ab,kf.
53. Mobile App*.ti,ab,kf. 54. phone*.ti,ab,kf.
54. phone*.ti,ab,kf.
55. smartphone*.ti,ab,kf. 56. cellphone*.ti,ab,kf. 57. mobile*.ti,ab,kf.
56. cellphone*.ti,ab,kf.
57. mobile*.ti,ab,kf.
58. web*.ti,ab,kf.
59. internet.ti,ab,kf.
60. app*.ti,ab,kf.
61. laptop*.ti,ab,kf.
62. video gam*.ti,ab,kf.
63. 1 or 2 or 3 or 4 or 5 or 6 or 7 or 8 or 9 or 10 or 11 or 12 or 13 or 14
64. 15 or 16 or 17 or 18 or 19 or 20 or 21 or 33 or 47
65. 22 or 23 or 24 or 25 or 26 or 27 or 28 or 29 or 30 or 31
66. 34 or 35 or 36 or 37 or 38 or 39 or 40 or 41 or 42 or 43 or 44 or 45 or 46 or 48 or 49 or 50 or 51 or 52 or 53 or 54 or 55 or 56 or 57 or 58 or 59 or 60 or 61 or 62 67. 63 and 64 and 65 and 66
67. 63 and 64 and 65 and 66
68. Chronic Disease/
69. chronic condition*.ti,ab,kf.

70. chronic disease*.ti,ab,kf. 71. chronic health.ti,ab,kf. 72. chronic illness.ti,ab,kf. 73. Long-Term Care/
74. Disease Management/ 75. ASTHMA/ 76. asthma.ti,ab,kf.
77. Bronchial Spasm/
78. Cystic Fibrosis/
79. Cystic Fibrosis.ti,ab,kf.
80. DIABETES MELLITUS/
81. diabetic.ti,ab,kf.
82. diabetes.ti,ab,kf.
83. OBESITY/
84. obesity.ti,ab,kf.
85. obese.ti,ab,kf.
86. obese.ti,ab,kf.
87. overweight.ti,ab,kf.
88. overweight.ti,ab,kf.
89. Attention Deficit Disorder with Hyperactivity/
90. Attention Deficit Disorder.ti,ab,kf.
91. ADHD.ti,ab,kf.
92. Cerebral Palsy/
93. Cerebral Palsy.ti,ab,kf.
94. Mental Disorders/
95. Mental Disorder*.ti,ab,kf.
96. mood disorder*.ti,ab,kf.
97. psychological disorder*.ti,ab,kf.
98. psychiatric disorder*.ti,ab,kf.
99. anxiety.ti,ab,kf.
100. depressive.ti,ab,kf.
101. depression.ti,ab,kf.
102. mental illness*.ti,ab,kf.
103. Chronic Pain.ti,ab,kf.
104. 68 or 69 or 70 or 71 or 72 or 73 or 74 or 75 or 76 or 77 or 78 or 79 or 80 or 81 or 82 or 83 or 84 or 85 or 86 or 87 or 88 or 89 or 90 or 91 or 92 or 93 or 94 or 95 or 96 or 97 or 98 or 99 or 100 or 101 or 102 or 103
105. 67 and 104
106. limit 105 to yr="2014 -Current"
107. remove duplicates from 106
