# Supplementary material for: Digital Behavior Change Interventions for Younger Children With Chronic Health Conditions: Systematic Review
Source: J Med Internet Res. 2020 Jul 31;22(7):e16924. doi: 10.2196/16924 (PMC7428934; doi:10.2196/16924)
Supplement: Multimedia Appendix 2 [file jmir_v22i7e16924_app2.docx]

## Multimedia Appendix 2: Full data extraction table

| **Reference** | **Population Characteristics** | | **Intervention characteristics** | | | | **Effectiveness** |  |
| --- | --- | --- | --- | --- | --- | --- | --- | --- |
|  | **Condition** | **Age** | **Overview of aims** | **Overview of Intervention** | **Overview of comparator** | **Theory/ modality** | **Behaviour Change Outcome**  Behaviour: measure: timepoint: results | **Primary Outcome (if different)** |
| \| **Quite promising** \| \| --- \| | | | | | | | | |
| Staiano et al., 2018 [61] | Overweight/ obesity | 10-12  (KS2 & KS3) | To test the effectiveness of the exergaming intervention to reduce adiposity and improve cardiometabolic health in children with overweight and obesity. | Digital component:  Participants were provided a Kinect® and Xbox 360® gaming console and four exergames (Your Shape: Fitness Evolved 2012, Just Dance 3, Disneyland Adventures and Kinect Sports Season 2) and a Fitbit Zip to wear during the 24-week period. Steps per day were wirelessly uploaded and reviewed by the fitness coach.  Other component:  The telehealth component consisted of the child and a parent meeting with a fitness coach over video chat, on a weekly basis for the first 6 weeks and biweekly thereafter. | Participants were asked to maintain their normal level of physical activity for 24 weeks and were provided the Xbox console and exergames following their final clinic visit. | Social Cognitive Theory,  First Wave (BT) | Physical activity:  ActiGraph GT3X+ accelerometer:  24 weeks:  The intervention showed an increase in more moderate/ vigorous physical activity (MVPA) compared with the control group. Intervention mean difference from baseline to follow-up = 3.6 MVPA minutes per day [SE 3.4] vs. Control mean difference= - 7.8 MVPA minutes per day [SE 3.2], *P* = .028.  Estimated mean difference from baseline to follow-up between intervention and control 11.4 MVPA minutes per day (95% CI: 2.25, 20.55).  Dietary habits:  The National Cancer Institute’s Self-administered 24-hr Dietary Recall:  24 weeks:  There was no difference in the change in total caloric intake between intervention and control. Intervention mean difference from baseline to follow-up = 297 calories [SE 215] vs. Control mean difference = 279 calories [SE 200], *P* = .069).  Estimated net mean difference from baseline to follow-up between intervention and control 18 calories (95% CI: -557.45, 593.54). | Body fat based on height and weight:  BMI Z-score:  24 weeks:  There was no clear evidence of effect on BMI z-score.  When the outlier was excluded compared with the control group. Intervention mean difference from baseline to follow-up = - 0.06 [SE 0.03] vs. control mean difference= 0.03 [SE 0.03], *P* = .016.  Estimated net mean difference from baseline to follow-up between intervention and control BMI z-score of -0.09 (95% CI: -0.17, 0.00).  In the intent-to-treat analysis, the Intervention mean difference = -0.06 [SE 0.03] vs. control mean difference= 0.02 [SE 0.03], *P* = .065).  Estimated net mean difference from baseline to follow-up between intervention and control BMI z-score of -0.08 (95% CI: -0.16, 0.003). |
| Trost et al., 2018 [62] | Overweight/ obesity | 8-12  (KS2 & KS3) | To evaluate the effects of incorporating active video gaming into an evidence-based paediatric weight management program | Digital component:  Participants were provided a game console and motion capture device (Xbox and Kinect; Microsoft Corporation) and 1 active sports game (Kinect Adventures!; Good Science Studio, Microsoft Game Studios) at their second treatment session. A second active game (Kinect Sports; Rare, Microsoft Game Studios) was provided in week 9 of the program. No explicit advice or goals were given to any study participant regarding the use of their active gaming tool.  Other component:  Participants were offered a comprehensive family-based paediatric weight management program. | Comprehensive family-based paediatric weight management program. | No theory reported,  First Wave (BT) | Physical activity:  accelerometer based motion sensor (GT3X or GT3X+; ActiGraph):  16 weeks:  The intervention group exhibited a greater increase in moderate/ vigorous physical activity (MVPA) compared with the control group.  Net mean difference from baseline to follow-up between intervention and control= 8.0 MVPA minutes per day (SE 3.8; 95% CI: 0.5, 15.4]; *P* = .04).  The intervention group also exhibited a greater increase in vigorous activity compared with the control group. Net mean difference= 3.1 minutes per day (SE 1.3; 95% CI: 0.6, 5.8]; *P* = .02). | The primary outcome was physical activity (see behavioural outcomes column). |
| Ahmad et al., 2018  [59] | Overweight/ obesity | 8-11  (KS2) | To evaluate the effectiveness of using social media and face-to-face sessions in a family-based intervention for weight management | Digital component:  Two training units delivered weekly via Facebook. In addition, weekly one-hour sessions using parents’ dedicated WhatsApp group that lasted for 12 weeks. In this phase the first author posted on WhatsApp key information and skills provided in the training phase, responded to any queries by parents and provided feedback on the adiposity progress of the children based on the measurements taken. Parents were encouraged to enquire and discuss with the researcher and to interact with other parents in the intervention group in order to promote programme adherence and maintain motivation using this platform.  Other component:  Two training units were delivered through half-day face-to-face sessions. | Waitlist control. | Social Cognitive Theory,  First Wave (BT) | Healthy lifestyle behaviours:  6 months:  effectiveness data not reported  Children’s eating:  behaviours, food and beverages consumption  6 months:  effectiveness data not reported. Paper currently under review.  Physical activity:  6 months:  effectiveness data not reported  Screen time:  6 months:  effectiveness data not reported | Body fat based on height and weight:  BMI Z-score:  6 months:  The intervention group had a reduced BMI z-score compared with the control group. Net mean difference from baseline to follow-up between intervention and control = −0.14 (95% CI: - 0.278, - 0.003), *P* = .05) |
| Jolstedt, et al., 2018 [60] | Anxiety | 8-12  (KS2 & KS3) | To assess the clinical efficacy and cost-effectiveness of a therapist-guided Internet Cognitive Behavioural Therapy (ICBT) protocol for children with anxiety disorders compared with an active control condition (internet-delivered child-directed play) | Digital component:  Internet-delivered CBT (ICBT). Web-based treatment programme, 12 modules presented over 12 weeks, consisted of texts, films, illustrations, and exercises. Limited weekly asynchronous support from a clinician, instructed to encourage and support, answer questions, help with troubleshooting, clarify the rationale for treatment, refer back to treatment content, and prompt and remind participants to log in and work with the programme. Parents work together with the child through the various treatment modules. Treatment focuses mainly on exposure therapy. Parents access 12 separate parent-directed modules in which they learn how parental behaviours can maintain anxiety and how to best support their child through treatment. Child- directed modules were directed to the child but the parent was present, and helped the child work with and complete these modules. | Internet delivered child-directed play.  Completely web-based treatment. Directed to the parent, and based on a parent directed intervention for children with behavioural problems, intended to strengthen the parent– child relationship. | CBT, Second Wave | None reported | Anxiety:  Clinician Severity Rating (CSR) derived from the Anxiety Disorder Interview Schedule for the DSM-IV:  12 weeks:  Participants allocated to ICBT showed improvements on the CSR compared to the active control. Net mean difference from baseline to follow-up between intervention and control = 0·79, (95% CI: 0·42, 1·16; *P* = ·002); the estimated between- group effect size at 12-weeks post-treatment 0·77 (95% CI: 0·40, 1·15). |
| Vigerland et al., 2016 [63]  &  Vigerland et al., 2017 [64] | Anxiety | 8-12  (KS2 & KS3) | To evaluate the efficacy of Internet- delivered CBT (ICBT) for children with anxiety disorders compared to a waitlist control. | Digital component:  A treatment platform presenting content in 11 chapters or modules. The content was presented in a varied manner with reading material, films, animations, illustrations, and exercises. A combined parent-child intervention with seven of the modules aimed at the parent(s), containing information and instructions on how to help their child, and four modules addressed to the child. Child directed content was a shorter version of the parent directed in- formation, adapted to an appropriate level and including less text and more animations. Participants had online contact with an assigned psychologist/CBT-therapist through written messages and written feedback on worksheets. Three telephone calls were scheduled during treatment, and additional telephone calls were conducted if it was necessary to increase motivation or problem solve. | Waitlist control. | CBT, Second Wave | None reported | Anxiety:  Clinician Severity Rating (CSR) derived from the Anxiety Disorder Interview Schedule for the DSM-IV:  10 weeks:  The intervention group had a larger improvement on the CSR. Net mean difference from baseline to follow-up between intervention and control = -1.16 (95% CI: -0.77, -1.55). |
| **Possibly promising** | | | | | | | | |
| Hsieh et al., 2018 [66] | Cerebral Palsy | 5-10  (KS1 & KS2) | To investigate whether training with Personal Computer games leads to greater learning motivation, and improved balance control in children with cerebral palsy | Digital component:  A PC gaming platform. The participants stood in front of the platform and viewed a monitor that displayed one of a series of simulated tasks, such as hitting ground rats. The gaming platform handle was loaded, 0.5 to 2.5 lb. The load of the handle was adjusted based on the activity level of the child’s trunk movements. This PC gaming platform provided trunk movements in 3 directions: horizontal, vertical and multidirectional trunk movements. | The control group played the same games as the intervention, but using a computer mouse | No theory reported,  First Wave (BT) | None reported | Measures of postural balance:  There was only clear evidence of effect of the intervention on postural balance on two of the seven measures: CoP, AP sway and Berg Balance Scale (BBS) scores.  12 weeks:  CoP, AP sway:  Intervention pre-test mean 9.94 (SD 2.27), post-test mean 9.17 (1.69). Control pre-test mean 10.87 (SD 1.41), post-test mean 10.55 (1.29).  Estimated difference in post-test means between intervention and control: -1.38 (95% CI: -2.33, -0.42).  CoP, ML sway:  Intervention pre-test mean 6.62 (SD 0.69), post-test mean 6.35 (1.00). Control pre-test mean 7.13 (SD 1.08), post-test mean 6.72 (0.70).  Estimated difference in post-test means between intervention and control: -0.37 (95% CI: -0.92, 0.18)  CoP, Sway area:  Intervention pre-test mean 14.42 (SD 2.35), post-test mean 13.71 (2.24). Control pre-test mean 14.33 (SD 2.39), post-test mean 13.93 (2.12).  Estimated difference in post-test means between intervention and control: -0.22 (95% CI: -1.61, 1.17)  CoP, Sway velocity:  Intervention pre-test mean 4.04 (SD 0.45), post-test mean 3.56 (0.47). Control pre-test mean 3.69 (SD 0.37), post-test mean 3.67 (0.23).  Estimated difference in post-test means between intervention and control: -0.11 (95% CI: -0.35, 0.13)  Berg Balance Scale (BBS) scores  Intervention pre-test mean 44.74 (SD 2.75), post-test mean 48.81 (4.74). Control pre-test mean 44.39 (SD 2.33), post-test mean 45.37 (2.68).  Estimated difference in post-test means between intervention and control: 3.44 (95% CI: 0.99, 5.89)  Fullerton Advanced Balance Scale (FAB) scores  Intervention pre-test mean 21.32 (SD 1.47), post-test mean 23.41 (2.09). Control pre-test mean 22.07 (SD 2.23), post-test mean 22.25 (1.90).  Estimated difference in post-test means between intervention and control: 1.16 (95% CI: -1.04, 4.04)  TUG scores  Intervention pre-test mean 16.43 (SD 2.12), post-test mean 17.51 (1.70). Control pre-test mean 15.60 (SD 1.10), post-test mean 15.91 (1.87).  Estimated difference in post-test means between intervention and control: 1.6 (95% CI: -0.37, 3.69) |
| Wantanakorn et al., 2018 [67] | Preoperative anxiety | 5- 12  (KS1,  KS2 & KS3) | To investigate the effects of children-Friendly Hospital, a tablet App for lowering preoperative anxiety prior to bone marrow aspiration. | Digital component:  "Children-Friendly Hospital"... a tablet application for paediatric patients who need bone marrow aspiration procedures. It is used to provide medical information and as part of preprocedural preparation. … participants started with the cartoon about the procedure then played matching games and practiced the breathing exercise game to reduce anxiety.  Other component:  Usual bone marrow aspiration procedures. | Usual bone marrow aspiration procedures. | No  theory reported,  First Wave | None reported | Anxiety:  Preoperative anxiety assessment tool:  mYPAS:  Anxiety was lower in the intervention group versus the control. Intervention mean = 55.37 (SD 12.86; 95% CI: 30.16, 80.58) versus control mean= 63.08 (SD 13.05; 95% CI: 37.50, 88.66). Estimated difference means between intervention and control= -7.71 (95% CI: -14.27, -1.15). |
| Bul et al., 2016 [65] | ADHD | 8-12  (KS2 & KS3) | To examine the effects of “Plan-It Commander”, and Internet-based serious game intervention for children with ADHD to teach and reinforce daily life skills, such as time management, planning/organizing, and cooperation skills. | Digital component:  The serious game is an online adventure game (called Plan-It Commander) developed by health care professionals, researchers, and game experts in collaboration with parents and children with ADHD. Plan-It Commander is an online learning environment in which principles of behaviour therapy and game-based learning were combined. Plan-It Commander is a mission-guided game divided into 10 different missions and several side missions. Missions guide the player’s behaviour throughout the game as he or she follows the storyline and is asked to solve problems requiring specific skills addressing time management, planning/organizing, and prosocial behaviour. In addition to the mission-guided game, players could access a closed social community (called “Space Club”) to stimulate prosocial behaviour (e.g., helping other players, giving compliments).  Other component:  Treatment-as-usual. | Treatment-as-usual. | No  theory reported,  First Wave (BT) | Time Management:  11 item Time management questionnaire, parent and teacher reported:  10 & 20 weeks:  The intervention arm showed greater improvements in parent reported time management skills compared to the control arm. Intervention least square mean= 10.66 (SE 1.64; 95% CI: 7.42, 13.89) vs control least square mean= 4.68 (SE 1.72; 95% CI: 1.29, 8.07) *P* = .004. As well as teacher reported time management. Intervention least square mean = 5.30 (SE 1.32; 95% CI 2.70, 7.90) vs Control least square mean= -0.16 (SE 1.38; 95% CI: -2.88 , 2.56) *P* =.01).  Estimated net mean difference of parent reported time management = 5.98 (95% CI: 1.32, 10.64) and teacher time management as 5.46 (95% CI: 1.71, 9.20).  Planning/organisation:  The subscale Plan/Organize of the Behaviour Rating Inventory of Executive Function parent and teacher reported:  10 & 20 weeks:  There was no difference in planning/organizing skills between the intervention and control arm, both on parent reported (Intervention mean= 1.47 (SE 0.36; 95% CI 0.75, 2.18) vs Control mean = 0.64 (SE 0.38; 95% CI: -0.11, 1.39 , *P* =.07)) and teacher reported ((Intervention mean 0.78 (SE 0.38; 95% CI: 0.11, 1.44) vs Control mean = 0.14 (SE 0.35; 95% CI: -0.55, 0.84 , *P* = .13)).  Estimated net mean difference of parent reported time management = 0.83 (95% CI: -0.20, 1.86) and teacher time management as 0.64 (95% CI: -0.32, 1.60).  Social Skills:  The subscale of the Cooperation of the Social Skills Rating System (SSRS) parent and teacher reported:  10 & 20 weeks:  There were no differences concerning participants’ cooperation skills on parent reported. Intervention mean= 1.10 (SE 0.34; 95% CI: 0.43, 1.78) vs Control mean = 0.46 (SE 0.36; 95% CI: -0.25, 1.16 , *P* = .13) or teacher reported (Intervention mean =2.95 (SE 0.67; 95% CI: 1.64, 4.27) vs Control mean = 2.36 (SE 0.70; 95% CI: 0.98, 3.74 , *P* = .48)  Estimated net mean difference of parent reported time management = 0.64 (95% CI: -0.33, 1.61) and teacher time management = 0.59 (95% CI: -1.31, 2.49). | The three primary outcomes were:  Parent reported Time Management,  Parent reported Planning/organisation,  Parent reported Social Skills  (see behavioural outcomes column). |
| **Non-promising** | | | | | | | | |
| Christison et al., 2016 [69] | Overweight/ obesity | 8-12  (KS2 & KS3) | To investigate the effectiveness of exergaming in paediatric group weight management. | Digital component:  The Exergaming for Health Program is a community-based, multifaceted paediatric weight management program that includes one hour of weekly physical activity curriculum of group exergaming to foster participation and increase entertainment value for children who are more likely to have lower perceived athletic competence.  Other component:  Classroom curriculum. | Classroom curriculum. | Family Systems Theory & Social Cognitive Theory  , First Wave (BT) | Activity levels:  Yamax 200 pedometer:  6 months:  There was no difference in daily steps between intervention and control from baseline to 6 months. -7684 (95% CI: -17847, 2479) *P* = .13  Sedentary screen  time:  Validated child media questionnaire:  6 months:  Total after school and Saturday screen time hours each reduced by 1 hour in the intervention compared to the control group, but this was not statistically significant.  Diet:  Block alive food  frequency questionnaire:  6 & 12 months:  Control participants reported consuming 0.8 (SD 0.5) more servings of fruit/day (*p* < 0.01), and 7% (SD 8.4%) fewer carbohydrates than intervention participants (*P* < 0.05). There were no differences between the groups in consumption of sugar sweetened beverages, fat, vegetable, or dairy servings. Combined, both groups consumed fewer sugared beverages/day from baseline (22.7 [25.2 , 20.3] *P* = .03) and consumed fewer total calories/day (2228 [ 2356 , 299] *P*  < 0.01). | Body fat based on height and weight:  BMI Z-score:  6 months:  There was no difference in BMI between intervention and control. Intervention mean change= -0.06 (SD 0.12) vs control mean change= 0.00 (SD 0.09) , *P* = .07).  Estimated net mean difference between intervention and control = -0.06 (95% CI: -0.35, 0.23). |
| Armstrong et al., 2017 [68] | Overweight/ obesity | 5-12  (KS1,KS2 & KS3) | To test the feasibility and effectiveness of a Motivational Interviewing (MI)- informed text messaging intervention for child obesity. | Digital component:  Daily mobile device text messages, based on MI, delivered for 12 weeks The first prompt of the week encourages parents to identify and set a health goal for the family, by selecting a self-determined behavioural change. In a reply text, the investigators preferentially reinforce the goals that are most evidence based and likely to lead to child BMI reduction (sugar-sweetened beverage reduction, increased physical activity, eating meals at home, and increased vegetable consumption). The investigator then prompts the parent, using an MI scaling tool, to assess their confidence in meeting the selected behavioural goal. The first texting conversation of the week includes three delivered text messages and three parent replies. Subsequent daily texts that week prompt parents to self-monitor adherence to the goal, reflecting the scaled confidence response. The subsequent texts of the week include one to two delivered text messages and one to two parent responses. Each week for 12 weeks, parents are invited to choose a new goal, or to continue working on the present goal.  Other component:  Standard care, which included monthly lifestyle counselling visits by a physician and dietician. | Standard care, which included monthly lifestyle counselling visits by a physician and dietician. Text message reminders for the 3-month study outcomes visit. | Motivational Interviewing Theory, Second Wave | Child Nutrition Habits:  The food frequency questionnaire:  12 weeks:  There was no differences in sugar-sweetened beverage consumption between intervention and control (Intervention median change= -1.0 (IQR -1.5, 0.5) Control median change= 0.0 (IQR -1.5, 1.0), *P* = .32) , fruit servings (Intervention median change 0.0 (IQR 0.0, 1.0) Control median change 0.0 (IQR -0.5, 1.0), *P* = .51) , vegetable servings (Intervention median change 0.0 (IQR -1.0, 0.5) Control median change 0.0 (IQR -0.5, 1.0), *P* = .42) or sugar snacks (Intervention median change 0.0 (IQR -2.0, 0.3) Control median change 0.0 (IQR -1.0, 1.0), *P* = .18).  Activity Habits:  youth risk behaviour surveillance:  12 weeks:  There was no difference in moderate-vigorous physical activity between intervention and control (Intervention median change 10.0 (IQR -2.5, 30.00) Control median change 0.0 (IQR -30.0, 25.0), *P* = .72).  Screen time:  youth risk behaviour surveillance:  12 weeks:  There was no difference in screen time between intervention and control (Intervention median change -0.5 (IQR -2.0, 0.5) Control median change 0.0 (IQR -1.0, 1.0), *P* = .17). | Body fat based on height and weight:  BMI Z-score:  12 weeks  There was no difference in BMI between intervention and control (Intervention median change 0.1 (IQR 0.0, 0.2) Control median change 0.0 (IQR -0.1, 0.1), *P* = .2) |
| Sanchez et al., 2017 [70] | Social- emotional problems | 7- 11  (KS1 & KS2) | To test whether children with social skills challenges report better social– emotional skills knowledge and improved mental health out- comes as a result of playing a digital social skills game, Adventures aboard the S.S. GRIN. | Digital component:  A game that require children to apply specific social–emotional skills to solve social problems encountered in the game. For example, presenting three true- to-life situations for elementary school children: approaching an individual that appears easy to talk to, joining a group at a game in progress, and approaching small groups that appear less easy to talk to . Within each scenario, the player had choices such as approaching or avoiding; interrupting or waiting; and being passive, assertive, or aggressive. Feedback to choices and prompts to respond were presented at appropriate times. We chose a single-player format for to enable individualized feedback and play paths based on player choices, allow for control of the reactions of other characters in a game, create a safe environment in which to practice fledgling skills and avoid the possible iatrogenic effects of participants reinforcing negative behaviours in other children. Players begin by creating a customized avatar, and the game has a nautical adventure theme. The child’s avatar joins the crew and travels around an island to address plot conflicts and save friendship on the island. Lines of mystery and ‘‘cliff-hangers’’ are woven throughout the story to maintain engagement across episodes. | Waitlist control. | Developmental theory, First Wave (BT) | Bullying perpetration:  The Bullying Others Subscale of the California Bullying Victimization Scale:  9 weeks:  There was no difference in bullying perpetration between intervention and control. Mean intervention difference -0.894 (SE 0.67) vs mean control difference 0.647 (SE 0.64) *P* = .099  Estimated net mean difference = -1.54 (95% CI: -3.36, 0.28) | No primary outcome specified. |
| **unable to assess effectiveness** | | | | | | | | |
| Fiks et al., 2015 [72] | Asthma | 6- 12  (KS1,KS2 & KS3) | To test the feasibility, acceptability, and impact on clinical outcomes of an innovative, electronic health records (EHR)-linked patient portal with decision support directed at both families and clinicians on asthma outcomes. | Digital component:  MyAsthma provided decision support to clinicians and parents. The features of MyAsthma include identification of parents’ concerns and goals for asthma treatment; monthly tracking of symptoms, medication side effects, and progress toward goals; asthma educational content including videos; and access to the child’s asthma care plan. Parents were encouraged with E-mail reminders to complete monthly portal surveys with input from their affected child. In response to these surveys, families and clinicians received guideline-based decision support that directed them to speak to one another if asthma was not well-controlled or if there were side effects, or to continue current therapy. Survey results were tracked over time in a timeline available to families through the portal and to clinicians through the EHR. | Standard care. Clinicians had access to a clinician- focused decision support system proven effective in fostering guideline-based care. | No  theory reported,  First Wave (BT) | Only acceptability/ feasibility data.  No effectiveness behavioural outcomes. | Only acceptability/ feasibility data.  No effectiveness behavioural outcomes. |
| Price et al., 2015 [75] | overweight/ obesity | 6- 12  (KS1,KS2 & KS3) | To describe the development and implementation of an interactive text messaging campaign for parents to support behaviour change among children participating in a childhood obesity intervention. | Digital component:  Text messages to parents to reinforce telephone health behaviour coaching. Text messages focused on primary target behaviours, including limiting fast food and eating fruits and vegetables in place of high calorie snacks. Text messaging schedule included: behavioural self-monitoring message followed by a skills training message about that same behaviour. Each skills training message offered a tip to encourage behaviour change. For example, ‘‘Most juices and sports drinks are loaded with sugar and calories—even 100% juice is. When your child is thirsty, water should be the drink of choice.’’ A conversational style was used.  There was an additional digital component; an alert to paediatricians at the time of a well child care visit, designed to identify children with a BMI ≥ 95th percentile. The alert contains links to the CDC growth charts, existing childhood obesity evidence, and a pre-populated, SmartSet® standardized well child visit template specific for obesity that includes: 1) instructions for documentation BMI percentile and diagnosis of obesity 2) documentation of  nutrition and physical activity counselling, 3) placing referrals for weight management programs, 4) placing orders for obesity-related laboratory studies and 5) links to printable patient education information and to a study website with additional obesity-related educational materials.  Additional component:  Well child visit. | This was a three-arm RCT. The other two arms were:  1.Standard care: Well child visits and follow-up appointments. Generic health-related materials in the mail.  2.Computerised decision support tools for clinicians, without the digital intervention for families. | Social Cognitive Theory, Second Wave (BT) | Only acceptability/ feasibility data.  No effectiveness behavioural outcomes. | Only acceptability/ feasibility data.  No effectiveness primary outcomes. |
| Kassee et al., 2017 [74] | Cerebral Palsy | 7- 12  (KS1,KS2 & KS3) | To explore the differences between a Nintendo Wii intervention, to a more conventional therapy in the home environment. (1) determine whether there was improvement in upper limb function after the Nintendo intervention, comparable to that of single-joint resistance training (2) explore differences in compliance rates, motivation levels and intervention feasibility, for parents and participants. | Digital component:  Participants assigned to Wii training were given a Nintendo Wii U system, one Wii MotionPlus Remote controller, one Wii Nunchuck, and the Wii Sports Resort game, to be played at home. Participants were instructed to play their choice of games, approved by the researchers to promote higher upper-limb activity. Participants were instructed to play the Wii using their affected hand for at least 40 minutes each day, 5 days a week for 6 weeks (30 days). No adaptions were made to the Wii-mote.  Other component:  Parents supervised the sessions and recorded the time, duration and games completed in a logbook. Parents were also asked to encourage the child to use their spastic hand as much as possible, and recorded how much they used their spastic hand in the logbook.  The time and intensity of exercise was considered comparable across the treatment and control.. | Participants were given equipment and a series of 6 exercises to do at home. Parents supervised the sessions and recorded the time, duration and games completed in a logbook. Parents were also asked to encourage the child to use their spastic hand as much as possible, and recorded how much they used their spastic hand in the logbook. | Motivation Theory,  First Wave (BT) | Exercise compliance:  Daily logs were used to report date, time, duration and repetitions of exercise:  10 weeks:  Pilot study (n=6), not powered to determine effectiveness. | Upper limb quality of movement and functional ability:  Melbourne-2  ABILHAND-Kids  questionnaire,  Average maximal grip strength:  10 weeks:  Pilot study (n=6), not powered to determine effectiveness. |
| Preston et al., [75] | Cerebral Palsy | 5-12  (KS1,KS2 & KS3) | To investigate the benefits of computer-assisted arm rehabilitation gaming technology on arm function of children with cerebral palsy. | Digital component:  The computer-assisted arm rehabilitation gaming at the child’s home. Parents were asked to encourage their children to use the gaming technology for 30 minutes a day.  Other component:  A visit to carried out after three weeks, to offer encouragement to the children and to check the gaming technology system.  Usual follow-up treatment following spasticity treatment with botulinum toxin | Usual follow-up treatment following spasticity treatment with botulinum toxin | No  theory reported,  First Wave (BT) | None reported | Upper limb quality of movement and functional ability:  ABILHAND-Kids:  12 weeks:  *Pilot study (n=15), not powered to determine effectiveness |
| Burckhardt et al., 2018 [71] | Type 1 diabetes. | 8-12  (KS2 & KS3) | To investigate the use of continuous glucose monitoring trends to prevent hypoglycaemia during exercise in young children with type 1 diabetes. | Digital component:  The Dexcom G5Ò Mobile Continuous glucose monitoring system allows transmission of sensor glucose levels via Bluetooth to a mobile device that generates alerts. This information can be shared via ‘‘cloud’’ with up to five individuals, who are then able to remotely monitor the Continuous glucose monitoring reading of the user in real time along with the possibility to use individualized alerts. participants were able to see their sensor glucose levels in real time. | .No digital device. | No  theory reported,  First Wave (BT) | Number of exercise  interruption events (such as stopping to perform self-monitored blood glucose tests or ingest carbohydrate):  The intervention arm was associated with fewer exercise interruption events compared with the control group. Incident rate ratios 0.19 (95% CI 0.10, 0.37 , *P* <0.001).*  The frequency of  carbohydrate intake:  The intervention arm was associated with fewer carbohydrate intake events compared with the control group. Incident rate ratios 0.38 (95% CI 0.22, 0.65 , *P*= .001).*  Carbohydrate intake  in grams:  There was no difference in carbohydrate intake between intervention and control.*  *Pilot study (n=14), not powered to determine effectiveness | No primary outcome specified. |
| Hamilton-Shield et al., 2014 [73] | Overweight/ obesity | 5-11  (KS1& KS2) | To investigate the clinical effectiveness, acceptability and cost-effectiveness of using a computer device, Mandolean, to retrain pro-obesogenic eating behaviours (speed of eating and portion size determination) in obese children and their families as an adjunct to standard lifestyle education in primary care clinics. | Digital component:  Mandolean® teaches patients how to eat and recognise hunger and satiety. The patient puts a measured portion of food determined by a therapist on the Mandolean (scales and computer) which records and displays, in real-time graphics, the removal of food from the plate as the patient eats. This is compared and matched to a pre-set eating line on screen displaying the speed at which the therapist wants the patient to eat. Deviation from the training line by eating too quickly or slowly elicits a spoken request from Mandolean to slow down or eat faster. At regular intervals, the patient rates their level of fullness (satiety): from 0 (no satiety) to 100 (maximum satiety). Patient-rated satiety appears as a dot on screen yielding a ‘development of satiety’ curve allowing comparison of the development of fullness to a ‘normal’ fullness curve again pre-set on screen. During ‘Mandolean training’ the patient gradually adopts a more normal pattern of eating and satiety by following these training lines and curves  Other component:  Standard care comprising of dietary and activity advice by trained practice nurses. | Standard care comprising of dietary and activity advice by trained practice nurses. | No  theory reported,  First-wave (BT) | Change in eating  Speed and self-determined portion size: measured using a ‘blind’ Mandolean, which acted solely as a measuring device:  12 and 24 months:  *  Changes in physical  activity levels:  New Lifestyles NL-800/ Nl-2000i pedometers :  12 and 24 months:  *  Children’s diets:  Food frequency questionnaire:  12 and 24 months:  *  *None of the pilot trial objectives were met, so no full-trial effectiveness results | Body fat based on height and weight:  BMI Z-score:  12 months:  None of the pilot trial objectives were met, so no full-trial results |
