# Supplementary material for: Digital Behavior Change Interventions for Younger Children With Chronic Health Conditions: Systematic Review
Source: J Med Internet Res. 2020 Jul 31;22(7):e16924. doi: 10.2196/16924 (PMC7428934; doi:10.2196/16924)
Supplement: Multimedia Appendix 3 [file jmir_v22i7e16924_app3.docx]

## Multimedia Appendix 3: Summary of risk of bias assessment

|  | Domain 1: Randomisation Process | Domain 2: Deviations from intended Intervention | Domain 3: Missing Outcome Data | Domain 4: Measurement of the Outcome | Domain 5: Selection of the Reported Results | Sample Size | Overall Judgement |
| --- | --- | --- | --- | --- | --- | --- | --- |
| Quite Promising |  |  |  |  |  |  |  |
| Ahmad et al, 2018^59^ | Low | Low | Low | Low | Some concerns . | 134 | Low |
| Jolstedt et al., 2018^60^ | Some concerns | Some concerns | Some concerns | Low | Some concerns | 131 | Some concerns |
| Staiano et al., 2018^61^ | Some concerns. | Some concerns | Low | Low | Low | 46 | Low/ some concerns |
|  |  |  |  |  |  |  |  |
| Trost et al., 2018^62^ | Low | Some concerns | Low | Low | Low | 75 | Low |
|  |  |  |  |  |  |  |  |
|  |  |  |  |  |  |  |  |
| Vigerland et al., 2016^63^  &  Vigerland et al., 2017^64^ | Some concerns | Some concerns | Low | Some concerns | Low | 93 | Some concerns/ Low |
| Possibly Promising |  |  |  |  |  |  |  |
| Bul et al., 2016^65^ | Some concerns | Some concerns | Some concerns | High/ some concerns | High | 170 | Some concerns/ high |
| Hsieh et al., 2019^66^ | Some concerns | Some concerns | Low | Some concerns | High | 40 | Some concerns/ high |
| Wantanakorn et al., 2018^67^ | Some concerns | High | Low | High | High | 60 | High/ some concerns |
|  |  |  |  |  |  |  |  |
| Non Promising |  |  |  |  |  |  |  |
| Armstrong et al., 2017^68^ | Low | Some concerns | Some concerns | High | High | 101 | High/ some concerns |
| Christison et al., 2016^69^ | Low | Some concerns | High | High | Low | 84 | Some concerns / high |
|  |  |  |  |  |  |  |  |
| Sanchez et al., 2017^70^ | Some concerns | High | High | High | High. | 69 | High |
| No effectiveness data available | | | | | | | |
| Burckhardt et al., 2018^71^ |  |  |  |  |  | 14 |  |
| Fiks et al., 2015 |  |  |  |  |  | 60 |  |
| Hamilton-Shield et al., 2014^73^ |  |  |  |  |  | 61 |  |
| Kassee et al., 2017^74^ |  |  |  |  |  | 6 |  |
| Preston et al.,2016^75^ |  |  |  |  |  | 15 |  |
| Price et al., 2015 |  |  |  |  |  | 549 |  |
