# Supplementary material for: Digital Behavior Change Interventions for Younger Children With Chronic Health Conditions: Systematic Review
Source: J Med Internet Res. 2020 Jul 31;22(7):e16924. doi: 10.2196/16924 (PMC7428934; doi:10.2196/16924)
Supplement: Multimedia Appendix 4 [file jmir_v22i7e16924_app4.docx]

## Multimedia Appendix 4: The users experience and views on the digital intervention; raw qualitative data and themes

| **Reference** | **Overview of digital component** | **Qualitative methods: Participants** | **Qualitative data from parents** | **Themes** | **Qualitative data from children** | **Themes** |
| --- | --- | --- | --- | --- | --- | --- |
| Hamilton-Shield et al., 2014^73^ | A computer device, (Mandolean) to retrain pro-obesogenic eating behaviours | Focus groups:  Children and parents. | ‘it does make me stop him and sit him down and make him eat the breakfast, rather than in the car’ | Parental involvement | ‘I like eating from the plate’  ‘I like the electronic stuff’ | Child-centred design |
|  |  |  |  |  | ‘It’s really confusing’ | Child-centred design |
|  |  |  | ‘it’s just second nature really’ ‘It’s not a huge disruption or anything’ | Technological affordances and barriers | ‘I put my fingers in my ears’ [regarding voice commands] | Child-centred design (need for personalisation) |
|  |  |  | ‘You’ve got to faff around’  ‘I think it’s hard, certain meals you have are harder to try and use it’ | Technological affordances and barriers | ‘boring’ ‘annoying’ | Child-centred design |
|  |  |  | ‘It says please rate your fullness… I don’t know how much [child] actually understands’  ‘irritating and annoying’ | Child-centred design |  |  |
|  |  |  | ‘I think enthusiasm’s gone off. In the beginning it was a lot easier’ | Technological affordances and barriers |  |  |
| Armstrong et al., 2017^68^  Linked pilot study with qualitative methods:  Sharifi et al., 2013^83^ | A Motivational Interviewing (MI)- informed text messaging intervention for child obesity | Focus groups and follow-up interviews:  Parents | ‘I think some kids will listen to their doctor better than their parents’ | Connection with a health professional is important for engagement | N/A | |
|  |  |  | ‘I don’t have to try to find the information. It comes to me.”  'I think the pro is that text is brief’ | Technological affordances and barriers |  |  |
|  |  |  | ‘It should go back somehow to the pediatrician, some way, so that we’re almost held accountable during the visit.’ | Connection with a health professional is important for engagement |  |  |
|  |  |  | “I didn’t start to text until the last year or two.”  ‘“They should know who has the [unlimited text messaging] plan…’ | Technological affordances and barriers |  |  |
|  |  |  | ‘“What if...I get to pick [the topics]. Don’t send me information about this…Every once in a while, you can throw some in, but I really want to focus on these.” | Technological affordances and barriers (need for personalisation) |  |  |
|  |  |  | “If you are getting these once a week and now its six weeks later and you haven’t really gotten any information that’s interesting to you, then I think I would text stop.” | Technological affordances and barriers |  |  |
| Fiks et al., 2015^72^ | An, electronic health records (EHR)-linked patient portal with decision support directed at both families and clinicians on asthma outcomes. | Open-ended questionnaires: Parents | ‘Communication…has been much more convenient’ | Technological affordances and barriers  Connection with a health professional | N/A | |
|  |  |  | ‘very easy…to keep track of everything’ | Technological affordances and barriers |  |  |
|  |  |  | ‘made me more aware of how serious his asthma could get’ | Parental involvement |  |  |
